# A metagenomic approach to One Health surveillance of antimicrobial resistance in a UK veterinary centre

**DOI:** 10.1099/mgen.0.001471

**Published:** 2025-09-01

**Authors:** Linzy Elton, Alonso Dupuy Mateos, Siân Marie Frosini, Rosanne Jepson, Sylvia Rofael, Timothy D. McHugh, Emmanuel Q. Wey

**Affiliations:** 1 UCL Centre for Clinical Microbiology, University College London, London, UK; 2 Independent Veterinary Surgeon, London, UK; 3 Pathobiology and Population Sciences, Royal Veterinary College, London, UK; 4 Clinical Science and Services, Royal Veterinary College, London, UK; 5 Faculty of Pharmacy, Alexandria University, Alexandria, Egypt; 6 Department of Infection, Royal Free London NHS Foundation Trust, London, UK

**Keywords:** antimicrobial resistance (AMR), metagenomics, MinION, Nanopore, One Health, Oxford Nanopore Technologies, veterinary

## Abstract

There are currently no standardized guidelines for genomic surveillance of One Health antimicrobial resistance (AMR). This project aimed to utilize metagenomics to identify AMR genes present in a companion animal hospital and compare these with phenotypic results from bacterial isolates from clinical specimens from the same veterinary hospital. Samples were collected from sites within a primary care companion animal veterinary hospital in London, UK. Metagenomic DNA was sequenced using Oxford Nanopore Technologies MinION. The sequencing data were analysed for AMR genes, plasmids and clinically relevant pathogen species. These data were compared to phenotypic speciation and antibiotic susceptibility tests of bacterial isolates from patients. The most common resistance genes identified were *aph* (*n*=101 times genes were detected across 48 metagenomic samples), *sul* (84), *bla*_CARB_ (63), *tet* (58) and *bla*_TEM_ (46). In clinical isolates, a high proportion of isolates were phenotypically resistant to *β*-lactams. Rooms with the greatest mean number of resistance genes identified per swab site were the medical preparation room, dog ward and surgical preparation room. Twenty-four and four plasmids typically associated with Gram-positive and *Enterobacteriaceae*, respectively, were identified. Sequencing reads matched with 14 out of 22 (64%) of the phenotypically isolated bacterial species. Metagenomics identified AMR genes, plasmids and species of relevance to human and animal medicine. Communal animal-handling areas harboured more AMR genes than areas animals did not frequent. When considering infection prevention and control measures, adherence to, and frequency of, cleaning schedules, alongside potentially more comprehensive disinfection of animal-handling areas, may reduce the number of potentially harmful bacteria present.

**Impact Statement**

It is becoming increasingly clear that antimicrobial resistance (AMR) is a problem that needs to be addressed with a multifactorial response, and recent studies have shown that both companion animals and the environment can be reservoirs for pathogens and AMR genes. The study of AMR within the veterinary hospital environment is limited, and, to our knowledge, this study is the first to use metagenomic methodologies to survey the veterinary hospital environment. This study demonstrates the importance of understanding environmental reservoirs in order to apply infection prevention and control policies effectively.

## Data Summary

All data are open access and are available in the supplementary materials. All sequencing data obtained in this study are available in the European Nucleotide Archive, under the project accession number PRJEB84924.

PubMed "veterinary" or "companion" AND "AMR" or "resistan*" NOT (Review[Publication Type]) (2018-2023).

"veterinary" or "companion" AND "AMR" or "resist*" AND "sequencing" or "metagenomic*" "veterinary" or "companion" AND "AMR" or "resistan*" NOT (Review[Publication Type]) (2018-2023).

https://www.iscaid.org/clinical-practice

## Introduction

Globally, many people live alongside companion or smallholding animals. In the UK, half of adults own a pet [1]. The concept of One Health (OH) has expanded the focus from human healthcare settings to include animal populations, wastewater and the environment as sources of antimicrobial resistance (AMR) transmission [2,4]. AMR was associated with 4.95 million human deaths in 2019, but data on the impact of AMR on companion animal morbidity and mortality are largely missing [5].

Despite the increasing popularity of companion animals, within the veterinary setting, AMR surveillance focus has been largely on food production animals. Multi-drug-resistant organisms (MDROs) such as methicillin-resistant staphylococci, extended-spectrum beta-lactamase (ESBL)-producing organisms and carbapenem-resistant *Enterobacterales* demonstrate resistance to multiple, important antibiotic groups. MDROs commonly cause human healthcare-associated infections (HHAIs) and are also increasingly being isolated from animals and the environment [6]. Companion animals such as dogs and cats are now considered to be AMR reservoirs [7,8].

Studies have shown that important MDROs such as *Escherichia coli*, *Staphylococcus aureus* and *Staphylococcus pseudintermedius* have been isolated from veterinary hospital environments around the world [9,11]. A recent systematic review of HHAIs and zoonotic MDROs stated that clinically relevant species were reportedly identified from environmental surfaces, clinical infections and fomites such as mobile phones and equipment such as hair clippers [11]. *Staphylococcus* species were the most reported organisms across the studies, followed by *E. coli*, *Enterococcus* spp., *Salmonella* spp., *Acinetobacter baumannii*, *Clostridium difficile* and *Pseudomonas aeruginosa* [11]. Veterinary healthcare workers, like their human healthcare counterparts, have also been found to be colonized with MDROs such as methicillin-resistant *S. aureus* (MRSA) [12]. Jobs involving animal handling are a risk factor for carriage of MDROs [11,13, 14]. There is also a greater risk of becoming infected (requiring treatment) with an MDRO if a host has been previously asymptomatically colonized [15]. Companion animals can become long-term carriers of MDROs following clinical infection and thus contribute to transmission within the animal, human healthcare and community environment [16,19].

Most of the previous literature looking at MDROs and AMR transmission in clinical veterinary and food chain environments has utilized phenotypic testing to identify both species and AMR. Several clinical studies in human hospitals have utilized genomics to show that AMR determinants and MDRO reservoirs can be found in healthcare setting environments, such as wastewater and from fomite swabbing, with similar data from food-animal production systems [20,24]. There are little data to show whether these AMR reservoirs are found in primary companion animal veterinary hospital environments.

Standardizing tools and guidelines for the genomic surveillance of OH AMR is complicated, owing to the varying pathogens, sample types and hosts [25]. In the UK, whilst there are human clinical and farm animal AMR surveillance programmes, there is no national surveillance programme for AMR in companion animals, and only limited data exist [26,27]. In this project, we aimed to utilize a metagenomic approach to identify AMR genes and clinically (both human and animal) relevant species present in a primary companion animal hospital environment and compare and relate them to phenotypic speciation and antibiotic susceptibility test (AST) results.

## Methods

### Clinical data collection

All samples and data were obtained from a primary care companion animal veterinary hospital in London, UK. Phenotypic pathogen species and AST data from all patient samples (dogs, cats and rabbits) submitted for bacteriology and susceptibility testing between January and December 2022 were obtained from the veterinary practice clinical records. Results obtained from the practice’s laboratory information management system (LIMS) included all samples irrespective of source (e.g. urine, bile, skin, wounds and faeces) submitted for bacteriology (IDEXX, Wetherby, UK). Bacterial speciation and MIC results, interpreted from CLSI VET01S ED5:2020 guidelines and using VITEK MS MALDI-TOF and VITEK-2 MIC, were extracted from the LIMS linked to IDEXX [28].

### Clinical data analysis

Clinical data were descriptively analysed to display the relationships between clinical isolate species, drug resistances seen and patient species.

### Environmental site selection and sample collection

For environmental sampling, a site visit to the veterinary hospital was undertaken prior to sample collection in order to evaluate room layout (Material S1, available in the online Supplementary Material), staff and patient routes of travel and determine prioritized sampling locations including both patient and non-patient areas (Material S2). Where there were multiple same-use rooms, e.g. consultation rooms, one representative location was chosen. Non-clinical rooms were also evaluated, including the visitor toilets. Samples were obtained on a normal working week afternoon shift in September 2022 from pre-designated locations. Sample types included wastewater samples (e.g. dog kennel waste drain), sponge swabs of surfaces (e.g. consulting tables), cotton stick swabs (e.g. sink drain holes) and liquid samples (e.g. ultrasound gel).

Further details of swabbing procedures for each swabbing site can be found in Material S3. For wastewater samples, 500 µl of wastewater was collected using a Pasteur pipette from the waste pipes of sinks, then placed into sterile screwcap tubes pre-dosed with 500 µl DNA/RNA Shield (Zymo Research Corporation). Cotton-tipped stick swabs (SS352, Appleton Woods) pre-moistened with 1× PBS (J61196.AP, Thermo Scientific) and stored in 500 µl PBS and 500 µl DNA/RNA Shield, and sponge swabs (TS/15-B, Technical Service Consultants Ltd.), pre-dosed with neutralizer buffer [29], were used to collect samples from ~5 cm^2^ of hard surfaces. Stick swab samples were transferred to sterile universal tubes containing 500 µl of DNA/RNA Shield. Sponge swabs were stored in their accompanying pouches until processing in the laboratory, where the liquid was aseptically removed with a Pasteur pipette, and 500 µl was added to 500 µl DNA/RNA Shield. All samples were refrigerated (2–8 °C) within 2 h of collection and processed in the laboratory within 24 h.

### DNA extraction and quantification

DNA was extracted from all environmental samples using the ZymoBIOMICS^™^ DNA Miniprep Kit (Zymo Research Corporation) following the manufacturer’s instructions [30]. A sample of ZymoBIOMICS^™^ Microbial Community Standard (Zymo Research Corporation) was included as an extraction control, following the manufacturer’s instructions [31]. DNA was assessed for concentration using the Qubit^™^ dsDNA BR Assay Kit (Thermo Fisher). Nuclease-free water (Sigma-Aldrich) was run as a negative control alongside samples from DNA extraction through to sequence analysis.

### Library preparation

DNA libraries were prepared using the Oxford Nanopore Technologies (ONT) Rapid PCR Barcoding Kit (SQK-RPB004) with a DNA input of 1–5 ng and following the manufacturers’ instructions [32]. ZymoBIOMICS^™^ Microbial Community DNA Standards (Zymo Research Corporation) were included as a sequencing control, following the manufacturer’s instructions [33].

### Sequencing and basecalling

Libraries were loaded onto a flow cell, version R9.4.1 (ONT), using a MinION device and were sequenced for 72 h, using the default parameters on the MinKNOW software (v23.04.6). Basecalling was performed by Guppy integrated into the MinKNOW software [41], using the high-accuracy algorithm. A total of 48 independent samples were evaluated in 4 runs of 12 barcoded samples. Two samples obtained from ultrasound gel were combined (samples 46 and 47), and the sample obtained from petroleum jelly did not yield any DNA and therefore could not be sequenced.

### Sequencing data analysis

Of the 48 samples subjected to sequencing, nine did not yield any reads. FASTQ files were quality-checked using FastQC (v0.21.1) and MultiQC (v1.15) [34,35]. Barcodes were trimmed from the reads using Guppy (v6.5.7) [36]. Sample data were analysed for the presence of AMR genes using KmerResistance 2.2 (v02-2018) using the ResFinder (v4.2.5) and KmerFinder (version 2022-07-11) databases, with 70% identity threshold, using the ONT pipeline [37,39]. Plasmids were identified using PlasmidFinder 2.1 (v2.0.1), with a minimum identity threshold of 95% and a minimum coverage threshold of 60% [37,40]. Species analysis was undertaken using Kraken (v2.1.3), and the NCBI Standard RefSeq indexes Plus Protozoa and fungi (2022-06-07), which includes bacteria, viruses, fungi, archaea and the human genome [41,42]. Non-human mammalian genomes, including cat or dog genomes, were reported as unclassified. The default confidence levels (0.0) were used when running Kraken, as ONT data have lower quality scores than Illumina, and these were environmental samples. Sequence data were deposited in the European Nucleotide Archive under BioProject PRJEB84924 and outlined in Material S2. For analysis of clinical isolate AMR status, an isolate was deemed multi-drug-resistant (MDR) if it showed resistance to antibiotics in three or more antibiotic classes to which the bacterial species does not show intrinsic resistance [43,44]. Statistical testing was conducted using GraphPad (Prism) (v10.3.0). One-way ANOVA and Tukey’s multiple comparisons tests were applied to the metagenomic samples to compare location, usage type or sample type data. Statistical significance was described as anything with *P*<0.05.

## Results

### Clinical data

The AST and speciation results for 148 clinical bacterial isolates from 91 dogs, 56 cats and 1 rabbit were obtained (Table 1). Twenty-two bacterial species were identified, of which the most commonly isolated were *E. coli* (46 isolates), *S. pseudintermedius* (28 isolates), *P. aeruginosa* (13 isolates), *Enterococcus faecalis* (12 isolates) and *Pasteurella multocida* (10 isolates). From AST data, the mean frequency of resistances per isolate across all species was 2.6 (sd=3.4). The species with the highest mean number of resistances were *Staphylococcus saprophyticus* (12, sd=0.0), *Staphylococcus haemolyticus* (10, sd=0.0), *S. aureus* (9, sd=5.0), *Staphylococcus lentus* (now *Mammaliicoccus lentus*) [45] (9, sd=0.0) and *Morganella morganii* (7, sd=0.0). Those with the lowest mean resistances were *P. multocida*, *P. aeruginosa*, *Salmonella* spp. and *Serratia fonticola* (0, sd=0.0). *P. aeruginosa* had the highest number of mean intermediate resistances (1.2, sd=0.4).

**Table 1.** Bacterial isolates identified from clinical samples during the year 2022. Total number of isolates identified, number of isolates stratified by host species, mean number of resistances per isolate and mean number of intermediate resistances per isolate

EB, Enterobacteriaceae; ET, endotracheal; GN, Gram-negative; GP, Gram-positive; NEB, non-Enterobacteriaceae; NF, non-fermenter.

The clinical isolates were most commonly resistant to antimicrobials in the *β*-lactam group; 35 out of 148 (23.8%) were resistant to at least one *β*-lactam antibiotic (Table 2). The drugs with the highest percentages of isolate resistance were penicillin with 33 out of 61 isolates tested (54.1%), amoxicillin with 57 out of 119 isolates tested (47.9%), ampicillin with 56 out of 126 (44.4%), cephalexin with 31 out of 117 (26.5%) and cefovecin with 27 out of 128 (21.1%). Resistance was not identified when tested to florfenicol (0 out of 56), moxifloxacin (0 out of 6), mupirocin (0 out of 39) or rifampicin (0 out of 39).

**Table 2.** Phenotypic antibiotic sensitivity testing results from 148 bacterial isolates taken from veterinary patients between January and December 2022, tested using VITEK MIC testing and interpreted from CLSI VET01S ED5:2020 guidelines

Drug group	Antibiotic	Total tested	Resistant	Intermediate
Aminoglycoside	Amikacin	118	9 (7.6%)	0 (0.0%)
	Gentamicin	118	2 (1.7%)	2 (1.7%)
	Neomycin	110	5 (4.5%)	2 (1.8%)
*β*-Lactams	Penicillin	61	33 (54.1%)	0 (0.0%)
	Ampicillin	126	56 (44.4%)	0 (0.0%)
	Amoxicillin	119	57 (47.9%)	0 (0.0%)
	Cloxacillin	43	9 (20.9%)	0 (0.0%)
	Oxacillin	43	9 (20.9%)	0 (0.0%)
	Amoxicillin–clavulanic acid	129	18 (14.0%)	7 (5.4%)
	Cephalexin	117	31 (26.5%)	0 (0.0%)
	Cefovecin	128	27 (21.1%)	2 (1.6%)
	Cefpodoxime	64	7 (10.9%)	0 (0.0%)
	ESBL screen	50	1 (2.0%)	0 (0.0%)
Amphenicols	Chloramphenicol	125	9 (7.2%)	10 (8.0%)
	Florfenicol	56	0 (0.0%)	0 (0.0%)
Fluoroquinolones	Ciprofloxacin	141	5 (3.5%)	5 (3.5%)
	Enrofloxacin	142	5 (3.5%)	8 (5.6%)
	Marbofloxacin	142	7 (4.9%)	2 (1.4%)
	Moxifloxacin	6	0 (0.0%)	0 (0.0%)
	Pradofloxacin	115	4 (3.5%)	2 (1.7%)
Fusidic acid	Fusidic acid	39	5 (12.8%)	0 (0.0%)
Macrolides	Azithromycin	49	10 (20.4%)	0 (0.0%)
	Erythromycin	49	10 (20.4%)	0 (0.0%)
Lincosamides	Clindamycin	24	4 (16.7%)	0 (0.0%)
	ICR test	16	3 (18.8%)	0 (0.0%)
	Lincomycin	39	9 (23.1%)	0 (0.0%)
Sulphonamides	Potentiated sulphonamides	122	15 (12.3%)	0 (0.0%)
Tetracyclines	Tetracycline	79	16 (20.3%)	0 (0.0%)
	Doxycycline	85	19 (22.4%)	0 (0.0%)
	Minocycline	56	3 (5.4%)	0 (0.0%)
Mupirocin	Mupirocin	39	0 (0.0%)	0 (0.0%)
Nitrofuran	Nitrofurantoin	78	8 (10.3%)	3 (3.8%)
Polypeptide	Polymyxin B	13	0 (0.0%)	13 (100.0%)
Rifamycin	Rifampicin	39	0 (0.0%)	0 (0.0%)

ICR, inducible clindamycin resistance.

One of the 46 *E. coli* (2%) isolates obtained from a feline ear swab was identified as MDR by phenotypic AST and 1 out of 7 (14%) *P*. *mirabilis* isolates from a canine ear swab. Two of the four *S. aureus* isolates (50%) were classified as MRSA, identified from a canine nasal swab and a feline ventral wound. Methicillin-resistant *S. pseudintermedius* (MRSP) was identified in 4 out of 28 (14%) *S*. *pseudintermedius* isolates (three canine ear swabs and one canine wound swab), and a further 6 out of 28 (21%) were classed as MDR (one canine ear swab, two canine eye swabs, two canine skin swabs and one canine urine sample), albeit methicillin-susceptible. The single *S. saprophyticus*, from a feline urine sample, was classed as MDR. Single isolates of *M. lentus* (feline ear swab) and *S. haemolyticus* (canine carpal screws) were also considered as MDR. A breakdown of clinical AST results stratified by species can be found in Material S6. A list of innate or commonly described resistances for the clinical species analysed in this study can be found in Material S4.

When topical-only antibiotics were tested, 100% of *P. aeruginosa* showed intermediate resistance (13 out of 13 isolates) to polymyxin B. For fusidic acid, *S. haemolyticus* was 100% resistant (1 out of 1 isolate), 10% of *S. pseudintermedius* was resistant (3 out of 28 isolates) and 35% of *Staphylococcus schleiferi* (1 out of 4 isolates) was resistant. No resistance was seen against mupirocin.

### Metagenomic data

#### General statistics

Fifty samples were collected from the veterinary hospital environment (34 sponge swabs, 7 cotton stick swabs, 5 wastewaters and 4 liquid samples). Forty-eight samples were sequenced, ten of which yielded no mapped reads when run through Kraken 2. A total of 19 different swabbing sample types across 15 different hospital rooms were evaluated by metagenomic analysis. Four of the 48 (8.3%) metagenomic samples did not yield any sequencing data (0 kb), so they were not included in the proportion analysis (dog ward: sink plug hole and stick swab; cat ward: door handles and sponge swab; cat ward: computer terminal and sponge swab; and imaging room: new (unopened) ultrasound gel #2, liquid sample). Ten of the 48 (20.8%) metagenomic samples (including the four mentioned above) did not produce classified reads when mapped, so they were not included in further speciation analysis (Table S2). The nuclease-free water negative control did not yield any data, so it was not included in the analysis.

When the total number of sequencing reads was calculated per sample and stratified by location, the pharmacy had the highest mean number of reads (5,589,075, sd=7,648,964), followed by the medical preparation area (3,299,602, sd=4,727,990) and the surgical preparation area (3,049,427, sd=4,356,555). When stratified by sample type, bin swabs had the highest mean number of reads (3,931,918, sd=5,208,454), followed by consulting tables (3,491,689, sd=4,832,317) and sink plug holes (2,952,655, sd=4,290,089) (see Material S5). When the samples were stratified by usage type (wastewater, dog-only area or item, cat-only area or item, communal animal area or item and human-only area or item), the largest mean number of reads was found in wastewater samples (2,699,265, sd=3,781,160). There was no significant difference in the total number of sequencing reads when one-way ANOVA and Tukey’s multiple comparisons tests were applied to the location, usage type or sample type data.

**Fig. 1.**
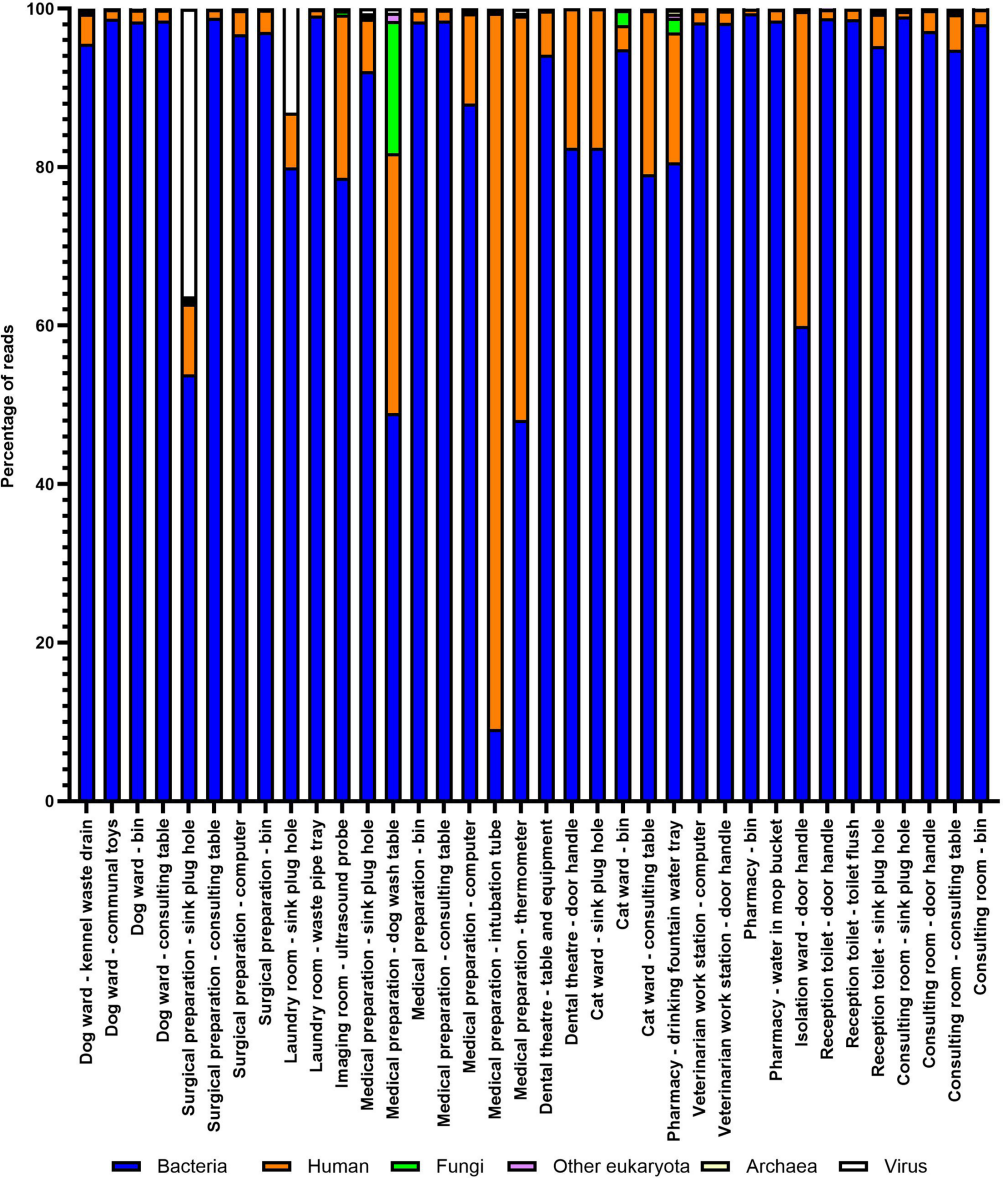
Breakdown of taxonomic classification of metagenomic sample reads. Sample sites that resulted in zero reads were not included in this figure.

When the total number of reads was classified taxonomically into bacterial, human, other eukaryotic, fungal and viral reads, 33 out of 36 (91.6%) samples had>50% bacterial reads (see Fig. 1). Sites with <50% bacterial reads were the medical preparation area dog wash table (49.0% bacterial, 32.8% human, 16.7% fungal, 1.0% other eukaryote, <0.1% archaea and 0.6% viral), the medical preparation area intubation tubes (10.0% bacterial, 90.3% human, 0.2% fungal, 0.2% other eukaryote and <0.1% archaeal and viral) and the medical preparation area thermometers (48.1% bacterial, 51.0% human, 0.1% fungal, 0.1% other eukaryote and <0.1% archaeal and viral). The sample site with the highest proportion of reads identified as fungi was the medical preparation area dog wash table (16.7% of reads). The sample site with the highest proportion of reads identified as viral was the surgical preparation area sink plug hole (36.3% of reads).

#### AMR gene identification

Across the total KmerResistance database of a possible 1,381 AMR genes, the most commonly identified AMR genes across the metagenomic samples were *aph*, conferring aminoglycoside resistance (genes were identified 101 times across all metagenomic samples), *sul* conferring sulphonamide resistance (84 genes), *bla*_CARB_ conferring *β*-lactam resistance (63 genes), *tet* conferring tetracycline resistance (57 genes) and *bla*_TEM_, also *β*-lactam resistance (46 genes). Ten *mecA* genes, 12 *blaZ* genes and 18 *blaOXA* genes were identified [46].

The rooms within the veterinary hospital in which the greatest number of AMR genes were identified were the medical preparation area (165 genes, sd=7.3), dog ward (136 genes, sd=6.6), surgical preparation area (63 genes, sd=3.3), veterinarian workstation room (50 genes, sd=2.3) and the consultation room (44 genes, sd=2.2) (Table 3). When stratified by sample type, the kennel waste drain (one sample, 64 genes) showed the greatest number of AMR genes. The consulting tables had the second highest mean (5 samples, mean=29, sd=6.2), then bins (6 samples, mean=20, sd=6.6), sink plug holes (7 samples, mean=16, sd=5.6) and seating areas (2 samples, mean=11, sd=1.9; Table 4).

**Table 3.** AMR genes stratified by room. Blue indicates that no AMR genes were found; the darker the red, the more genes were identified

**Table 4.** AMR genes stratified by sample type. Blue indicates that no AMR genes were found; the darker the red, the more genes were identified

#### Plasmid identification

Twenty-four plasmid types associated with AMR gene transmission in Gram-positives were identified, out of a total of 329 in the database [46]. The highest mean number of Gram-positive plasmids was found in the dog ward (mean=3.7), surgical preparation area (mean=1), consulting room (mean=0.8), medical preparation area (mean=0.6) and the cat ward (mean=0.2) (see Table 5).

**Table 5.** Gram-positive plasmids stratified by (a) room and (b) sample type. Blue indicates that no plasmids were found; the darker the red, the more genes were identified

Four plasmid types associated with AMR gene transmission in Enterobacterales were identified, out of a total of 159 in the database [46]. Col (pHAD28) was identified from the kennel waste drain swab in the dog ward, and Col440I, IncQ2 and IncP6 were identified from the sink plug hole swab from the medical preparation area (see Table 6). No other plasmids associated with Enterobacterales were identified.

**Table 6.** Enterobacterial plasmids stratified by (a) room and (b) sample type. Blue indicates that no plasmids were found, and red indicates that a plasmid was identified

#### Metagenomic speciation

Sequencing reads mapped with 14 out of 22 (64%) of the pathogenic species identified phenotypically from the clinical isolates. The species to which the highest overall mean number of reads mapped was *P. aeruginosa* (1,678,521 reads), *E. coli* (569,637 reads) and the coagulase-negative staphylococci (CoNS), for which reads mapped to *S. capitis*, *S. cohnii*, *S. epidermidis*, *S. equorum*, *S. felis*, * S. haemolyticus*, *S. hominis*, *S. saccharolyticus*, *S. saprophyticus*, *Mammaliicoccus sciuri* (formerly *Staphylococcus sciuri*), *S. succinus*, *S. warneri* and *S. xylosus* (547,367 reads), and the Moraxella group, for which reads mapped to *M. bovoculi*, *M. catarrhalis*, *M. cuniculi*, *M. nonliquefaciens*, *M. osloensis* and *M. ovis* (529,572 reads). No sequencing reads mapped to *Hafnia alvei*, *Klebsiella oxytoca*, * P. multocida*, *Proteus mirabilis*, *S. fonticola*, *S. auris*, *S. lentus* or *S. schleiferi*.

Four species (*E. faecalis*, *E. coli*, Moraxella group and *S. pseudintermedius*) in the clinically pathogenic samples were found across 7 out of 15 (46.7%) locations sampled. Three species (CoNS, *Klebsiella pneumoniae* and *P. aeruginosa*) were found in 6 out of 15 (40.0%) of locations. The medical preparation area had the highest number of reads mapping to species isolated from clinical samples (1,100,313, 30% of the total reads mapped to clinical species), followed by the laundry room (969,382, 26%) and dog ward (541,367, 15%). The backyard, cat reception area, dental theatre and dog reception area had no reads mapping to the clinical species (Table 7a).

**Table 7.** Relative abundance identified for each clinical species identified through culture and mass spectrometry: (a) relative abundance stratified by location and (b) relative abundance stratified by sample type. Species groupings include all species of that genus for which reads were identified. *Enterococcus* spp. (not *E. faecalis*): *E. casseliflavus*, *E. faecium*, *E. hirae* and *E. luminosum*. Moraxella group: *M. bovoculi*, *M. catarrhalis*, *M. cuniculi*, *M. nonliquefaciens*, *M. osloensi*s and *M. ovis*. CoNS: *S. capitis*, *S. cohnii*, *S. epidermidis*, *S. equorum*, *S. felis*, *S. haemolyticus*, *S. hominis*, *S. saccharolyticus*, *S. saprophyticus*, *S. succinus*, *S. warneri* and *S. xylosu*s. *Salmonella* spp.: *S. enterica*. Green indicates that no AMR genes were found, and yellow to red indicates increasing relative abundance

EB, Enterobacteriaceae; GN, Gram-negative; GP, Gram-positive; NEB, non-Enterobacteriaceae; NF, non-fermenter.

When stratified by sample type, sink plug hole swabs had the highest number of reads mapping to clinical species (1,842,605, 51% of the total reads mapped to clinical species), followed by the consulting tables (698,536, 19%) and bins (366,020, 10%). The dog washing table, intubation tubes, ultrasound gel, backyard wastewater pipe, petroleum jelly, seating areas, dental surgery table and equipment and toilet flushes had no reads mapping to the clinical species. Ninety-seven per cent of reads mapping to *P. aeruginosa* were found in sink plug holes (Table 7b). The Moraxella group was found in 7 out of 20 (35.0%) sample types. *E. faecalis*, *E. coli*, *P. aeruginosa* and * S. pseudintermedius* were found in 6 out of 20 (30.0%) sample types, and CoNS and *K. pneumoniae* were found in 5 out of 20 (25.0%).

## Discussion

### Presence of clinically relevant AMR genes in the veterinary hospital environment

There is an increasing awareness of the role of the companion animal/owner interaction in the transmission of AMR. Here, we provide evidence that the veterinary hospital environment mirrors human hospitals in that they may act as a reservoir for infectious agents and drug-resistant organisms in particular. AMR genes that were consistent with the phenotypic clinical isolate resistance profiles were found in the environmental samples, and areas with the most AMR genes were areas with high levels of animal handling such as the preparation rooms, wards and consultation room, indicating a route for transmission. Bins, consulting tables and sink plug holes were the sample types with the highest numbers of AMR genes identified, also suggesting a link between AMR gene reservoirs and areas with high animal contact and waste disposal.

Ten *β*-lactam resistance genes were identified in the metagenomic samples, and this was echoed in the resistances seen in the clinical isolates. *β*-Lactam antibiotics are clinically important in both animal and human medicine, although access to many of the antimicrobials used to treat ESBL infections in the human sector is not available in veterinary settings [47]. Third-generation cephalosporins are not commonly used in small animal veterinary centres in the UK, although this may vary from clinic to clinic [48]. Sulphonamide and tetracycline genes were commonly found in the environmental samples (in 8 and 11 of the sampling areas, respectively), but few clinical isolates showed resistance to these drugs. *mecA* genes were identified in the metagenomic samples, and whilst they are found in a number of staphylococcal species, MRSP has been commonly isolated from dogs, including being carried on the skin sub-clinically and long term following disease resolution, with the potential for dissemination into the environment through natural desquamation [49]. MRSP has been increasingly isolated from humans, especially those with close contact with dogs, such as veterinarians and dog owners [50,51].

There is increasing evidence that companion animal microbiomes may shape their owners’ AMR microbiomes, with the potential for both pathogens and AMR genes and other mobile genetic elements capable of being transmitted between pets and their owners [52]. Increasing numbers of studies have highlighted risk factors for the spread of AMR and pathogens within a companion animal household, such as previous veterinary hospital visits and feeding regimens, with raw food being a particular risk [53,55]. Less studied is the role of the veterinary environment as a reservoir in the transmission of pathogens and AMR. Here, we aimed to compare the clinical infection data from companion animals over the previous year, with a metagenomic snapshot view of the veterinary centre environment. This study did identify AMR genes in the veterinary environment that were associated with the resistance seen in the clinical cases, suggestive of potential environmental reservoirs and the possibility that veterinary hospitals may shape the resistomes of pets and therefore potentially their guardians. However, metagenomics is not sensitive enough to identify causal links, and even higher-resolution sequencing, such as whole-genome sequencing, requires detailed clinical metadata such as patient movements, dates of hospitalization and background risk factor analyses to robustly ascertain transmission of AMR.

### Identification of plasmids associated with AMR gene carriage

Plasmids that can carry AMR genes are an OH concern, as they can be easily spread between bacteria, both within and between hosts, and in the environment [56]. Most Gram-positive plasmids identified were linked to *Staphylococcus* species and were most commonly recorded in the dog-related areas, such as the dog ward, and from bin samples, suggesting that they may be *S. pseudintermedius*-mediated. Many identified contained *erm* genes for erythromycin resistance (including pDLK1, pMSA16, pNE131, pRUM, pWBG738 and SAP085B), and nearly a third of the *S. pseudintermedius* phenotypically tested were resistant to erythromycin [57,62]. A similar level of resistance to the lincosamides was seen in *S. pseudintermedius*, and pLNU4, encoding *lnu* genes for lincomycin resistance in staphylococci, was the most commonly identified plasmid [63]. pSK108 was the second most commonly identified plasmid and, along with pWBG574, encodes *qacC*, a multi-drug (β-lactam) resistance gene in staphylococci, which may account for some of the *β*-lactam resistance seen, alongside *mecA*, which is not plasmid-mediated, but found on the chromosomal *SSCmec* cassette [64,65].

Plasmids from the Enterobacteriales were much less commonly identified from the environmental samples, and the clinical enterobacterial isolates generally showed lower rates of phenotypic resistance compared with the staphylococcal isolates, but all four identified are implicated in the carriage of AMR genes [66,69]. Col (pHAD28) plasmids harbour *qnrB* genes, conferring resistance to quinolones, and low numbers of isolates had phenotypic quinolone resistance [70]. Col440I can harbour carbapenemase genes such as *bla*_NDM-1_, whilst IncQ2 encodes *bla*_CMY-4_, *bla*_GES-1_ and the sul2-*strA-strB* gene cluster [67,71]. Whilst resistances to some of the *β*-lactam genes were common in some of the enterobacterial isolates, some drugs had low levels of resistance, suggesting a complex interplay of genetic factors. Low numbers of enterobacterial isolates had sulphonamide resistances, and streptomycin was not tested for any of the clinical isolates, likely because streptomycin is not commonly used clinically in companion animals, as per the prescribing cascade and due to its toxicity in small animals [48,72]. IncP plasmids have been reported to carry resistance genes for *β*-lactams, sulphonamides, aminoglycosides and tetracyclines, and recently, genes for colistin resistance, although no *mcr* genes were identified in this study [67].

### Presence of clinically isolated species in metagenomic samples

Almost two-thirds of the clinically isolated bacterial species were also found in the environmental metagenomic samples, and many are known to also be common environmental species, although pathogenic and non-pathogenic strains may differ phenotypically as a response to differing environmental stressors [73,76]. *E. coli* and *P. aeruginosa* were commonly identified as clinical isolates and also present within the environmental metagenomic samples. *S. pseudintermedius*, the second most frequently found clinical isolate, was commonly found across the locations and sample types in the veterinary hospital. As it was also found in the preparation rooms where both cats and dogs were handled, this could suggest cross-contamination. Alongside the clinically isolated species, other environmental, non-pathogenic species were also found. These organisms may also play a role in AMR transmission, as they are capable of harbouring plasmids, AMR genes and other mobile genetic elements [77].

### Implications for infection prevention and control

From an infection prevention and control (IPC) perspective, the results identified in this study point to some potential risk factors. The use of communal items, such as dog toys, may pose a risk of infection and/or AMR transmission. Whilst a more intensive cleaning schedule could be suggested, in reality, it may be impossible in a busy practice to implement more regular cleaning, and therefore, a combination of IPC measures may be needed and a reduction in the use of communal/‘home’ animal patient items. Similarly, the high number of AMR genes found in the consulting table swabs suggests the potential for transmission, especially if they are used continually and not cleaned immediately after use. The non-slip rubber surface on these tables was observed to be covered in small scratches, presumably from animal claws, which would make them difficult to comprehensively clean. An alternative, more robust non-slip material could be utilized. The bin, kennel waste drain and sink plug hole samples also had high numbers of AMR genes identified. These data may suggest a call for more regular, or more thorough, deep cleaning of these areas, to reduce the risk of transmission to both humans and animals. The use of foot-pedal-operated bins to reduce the risk of hand transmission, and the consideration of bin aerosols with swing top bins, should also be taken into account.

Hospital areas with fewer AMR genes identified were those with lower or no animal presence and/or movement, such as the pharmacy, imaging room and isolation ward, although as sampling was done at a single time point, the effect of before and after cleaning could not be elucidated. Another area of low animal movement and no AMR genes recovered was the cat waiting area. In comparison, 22 AMR genes were identified in the sample from the dog waiting area. This may be due to the fact that cats will likely be in cat carriers whilst waiting, whereas dogs will be on the floor, or on the seats. With a large volume of pets waiting in these areas, cleaning cannot be undertaken between every patient, which strengthens the argument that high animal-movement areas, especially of dogs, are likely to have more AMR genes present in the environment, making them higher transmission risk areas.

The clinical pathogens most commonly identified within the environmental metagenomic samples were *E. faecalis*, *E. coli*, the Moraxella group, *S. pseudintermedius*, CoNS, *K. pneumoniae* and *P. aeruginosa*. These species can often harbour high numbers of resistance genes, as well as the ability to possess and transmit mobile genetic elements such as plasmids. Their presence in the veterinary hospital environment poses a potential risk for pathogen transmission and the provision of a reservoir for AMR genes of importance, such as *mecA*, which may circulate between non-pathogenic and pathogenic species.

### Use of genomic evidence for augmenting IPC

This study demonstrates that metagenomic sequencing analysis can be a useful tool for broad environmental surveillance of AMR genes and potentially pathogenic species and may be impactful in terms of identifying IPC risk and areas for intervention. Long-read technologies are especially useful in metagenomic surveillance when coupled with long-read analysis programmes, as even PCR-amplified sequences (in this study, the amplicons were around 4 kb) will span greater regions of genomes and plasmids, providing greater resolution when differentiating between species and AMR genes or SNPs. Whilst the cost may mean it is used in a targeted manner to guide practice rather than routinely, metagenomic surveillance for AMR in (human) clinical environments is gaining traction [78]. There are limitations, however, in terms of clinical aspects. The isolates sent for microbiological testing are more likely to have raised clinical concerns than those successfully treated empirically. For sampling, whilst we undertook sample acquisition on a random weekday, it did not take into account the cleaning schedule of the practice, which could have influenced the amount of DNA obtained from each area. Equally, we were not able to swab certain rooms as they were in use at the time, which may also have introduced bias, as certain areas may be more heavily used than others.

In terms of sequencing, a lack of sensitivity/resolution makes it difficult to identify more detailed relationships, such as which species carry which plasmids and non-chromosomal AMR genes, as well as the bioinformatics tools and databases used to identify genetic elements, which are continuously developing. This makes it difficult to identify whether it is the pathogenic or environmental/commensal species, with MDRO profiles. The number of reads is an imprecise measure of the quantity of DNA. However, it is a widely used metric to provide some indication of the number of bacteria present, and whilst the absolute numbers are likely to be inaccurate, comparative read numbers, especially when a control community is included, have proven to be a useful indicator of differences in load. Without specific enrichment or isolation of a species for whole-genome sequencing, it is also not possible to identify transmission patterns of potential healthcare-associated infections. However, the use of metagenomic sequencing can help to pinpoint areas of interest and thus reduce potential sampling and costs for secondary, more detailed studies. Further considerations are the difficulties in ensuring standardization of sample collection and the ability to process some sample types, such as alcohol- and oil-based gels, as they are not compatible with DNA extraction kits.

## Conclusions

The results of this study should be placed in context; the veterinary hospital environment is not anticipated to be sterile, and the identification of bacterial organisms including those with AMR genes, plasmids and MDRO genotypes is not unexpected. Whilst an MDRO genotype does not necessarily confer greater virulence, it can make organisms more challenging to treat. Our study suggested that high animal-handling and animal-movement areas, as well as areas in which dogs are present, are more likely to harbour AMR genes, plasmids and potentially pathogenic species. This information can be of clinical importance when considering IPC measures; however, these findings would support the consideration of risk-based assessments to determine the frequency of cleaning and disinfection protocols within given areas. Surfaces that come into contact with animals or animal fluids should be considered intervention targets, together with the importance of measures such as hand hygiene to reduce the risk of direct and indirect transmission within the hospital environment.

## Supplementary material

10.1099/mgen.0.001471 Uncited Supplementary Material 1.

## Abbreviations

AMR: antimicrobial resistance
AST: antibiotic susceptibility test
CoNS: coagulase-negative staphylococci
ESBL: extended-spectrum beta-lactamase
HHAI: human healthcare-associated infection
ICR: inducible clindamycin resistance
IPC: infection prevention and control
LIMS: laboratory information management system
MDR: multi-drug resistance
MDROs: multi-drug-resistant organisms
MRSA: methicillin-resistant *Staphylococcus aureus*
MRSP: methicillin-resistant *Staphylococcus pseudintermedius*
OH: One Health
ONT: Oxford Nanopore Technologies
