# Supplementary material for: A metagenomic approach to One Health surveillance of antimicrobial resistance in a UK veterinary centre
Source: Microb Genom. 2025 Sep 1;11(9):001471. doi: 10.1099/mgen.0.001471 (PMC12401494; doi:10.1099/mgen.0.001471)
Supplement: Uncited Supplementary Material 1. [file mgen-11-01471-s001.pdf]

Supplementary materials

1. Veterinary Hospital floor plan

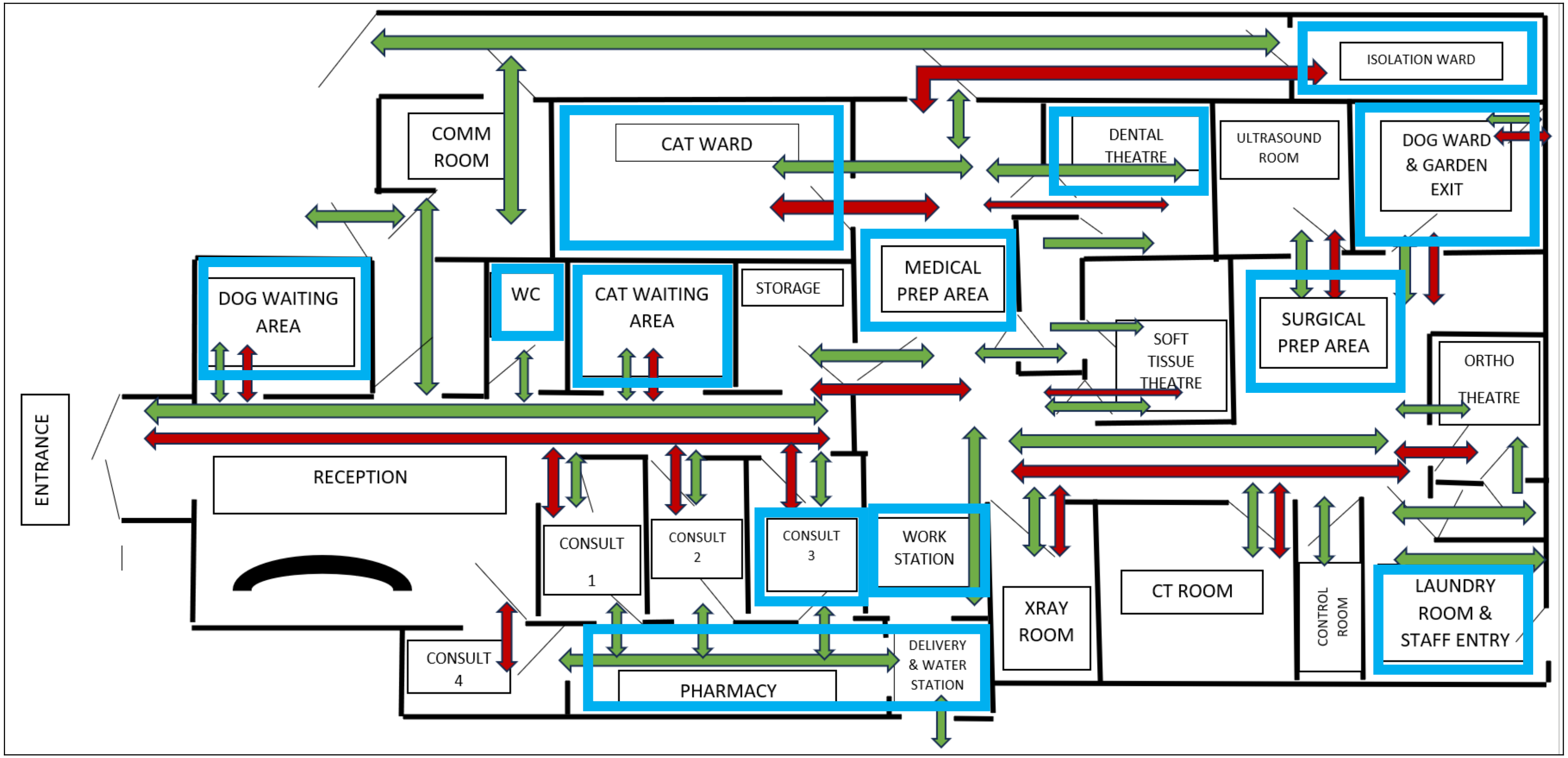

Layout of Small Animal Veterinary Hospital where samples were taken (not to scale). Green arrows represent human flow of movement. Red arrows show animal flow of movement. Areas highlighted by blue boxes were the rooms sampled.

2. Sample data and accession numbers

BioProject number: PRJEB84924

Sample number	Room	Description	Sample type	Total mapped reads	Fastq file size	Sample ID
1	Dog ward	Sink - plug hole	Stick swab	0	0 KB	No data, not uploaded
2	Dog ward	Kennel waste drain	Wastewater	1,349,483	961 MB	ERS22966141
3	Dog ward	Communal toys	Sponge swab	544,391	1.04 GB	ERS22966142
4	Dog ward	Computer terminal	Sponge swab	0	21.09 MB	ERS22966143
5	Dog ward	Bins	Sponge swab	981,862	698 MB	ERS22966144
6	Dog ward	Consulting table	Sponge swab	341,631	456 MB	ERS22966145
7	Surgical preparation area	Sink - plug hole	Stick swab	9,394,133	5.55 GB	ERS22966146
8	Surgical preparation area	Consulting table	Sponge swab	350,816	229 MB	ERS22966147
9	Surgical preparation area	Computer terminal	Sponge swab	52,939	239 MB	ERS22966148
10	Surgical preparation area	Bins	Sponge swab	2,399,821	2.66 GB	ERS22966149
11	Laundry room	Sink - plug hole	Stick swab	1,384,049	368 MB	ERS22966150
12	Laundry room	Waste pipe and water catch tray	Wastewater	1,116,071	598 MB	ERS22966151
13	Imaging room	Ultrasound probe	Sponge swab	5,974	6.18 MB	ERS22966152
14	Dog ward	Door handles	Sponge swab	31	<1 KB	ERS22966153
15	Back yard	Outside open water pipe	Wastewater	115	20.53 KB	ERS22966154
16	Medical preparation area	Sink - plug hole	Stick swab	8,990,190	5.77 GB	ERS22966155
17	Medical preparation area	Dog washing table	Sponge swab	290	1.64 GB	ERS22966156
18	Medical preparation area	Bins	Sponge swab	2,944,186	2.62 GB	ERS22966157
19	Medical preparation area	Consulting tables	Sponge swab	10,991,957	4.9 GB	ERS22966158
20	Medical preparation area	Computer terminal	Sponge swab	28,521	12.92 MB	ERS22966159
21	Medical preparation area	Intubation/oxygen tubes	Sponge swab	3,200	17.11 MB	ERS22966160

22	Medical preparation area	Thermometers	Sponge swab	138,872	78.31 MB	ERS22966161
23	Dental theatre	Table and equipment	Sponge swab	11,592	60.12 MB	ERS22966162
24	Dental theatre	Door handles	Sponge swab	62	374 KB	ERS22966163
25	Cat ward	Sink - plug hole	Stick swab	654,306	493 MB	ERS22966164
26	Cat ward	Bins	Sponge swab	367,562	1.32 GB	ERS22966165
27	Cat ward	Consulting table	Sponge swab	3,280	6.83 MB	ERS22966166
28	Cat ward	Door handles	Sponge swab	0	0 KB	No data, not uploaded
29	Cat ward	Computer terminal	Sponge swab	0	0 KB	No data, not uploaded
30	Pharmacy/drinking room	Drinking fountain water tray	Wastewater	2,027,394	3.17 GB	ERS22966167
31	Veterinarian workstation room	Computer stations	Sponge swab	2,176,452	845 MB	ERS22966168
32	Veterinarian workstation room	Door handles	Sponge swab	165,271	189 MB	ERS22966169
33	Pharmacy/drinking room	Bins	Sponge swab	14,369,377	3.44 GB	ERS22966170
34	Pharmacy/drinking room	Water in mop bucket	Wastewater	370,453	120 MB	ERS22966171
35	Isolation ward	Door handles	Sponge swab	41,283	22.92 MB	ERS22966172
36	Reception toilet	Door handles	Sponge swab	107,957	35.13 MB	ERS22966173
37	Reception toilet	Toilet flush	Sponge swab	6,611	3.42 MB	ERS22966174
38	Reception toilet	Sink - plug hole	Stick swab	190,649	264 MB	ERS22966175
39	Cat reception area	Seating area	Sponge swab	0	382 MB	ERS22966176
40	Dog reception area	Seating area	Sponge swab	56,017	136 MB	ERS22966177
41	Consulting room 3	Sink - plug hole	Stick swab	55,255	23.45 MB	ERS22966178
42	Consulting room 3	Computer terminal	Sponge swab	0	107 KB	ERS22966179
43	Consulting room 3	Door handles	Sponge swab	29,839	16.58 KB	ERS22966180

44	Consulting room 3	Consultation tables	Sponge swab	5,770,761	3.01 GB	ERS22966181
45	Consulting room 3	Bin	Sponge swab	2,528,700	1.02 GB	ERS22966182
46+47	Imaging room	Old (in use) ultrasound gel	Liquid sample	0	11.97 KB	ERS22966183
48	Imaging room	New (unopened) ultrasound gel #1	Liquid sample	0	43.73 KB	ERS22966184
49	Imaging room	New (unopened) ultrasound gel #2	Liquid sample	0	0 KB	No data, not uploaded
50	Imaging room	Petroleum jelly	Liquid sample	0	33.63 KB	ERS22966185

3. Swabbing procedures

Sample type	Swab type	Size/volume	Process
Bins	Sponge swab	~5 cm^2	The lid and front of the bins were swabbed (sampling areas were selected at random)
Communal toys	Sponge swab	~5 cm^2	A sampling area was selected at random, from a toy that was also selected at random from the box
Computer terminal	Sponge swab	~5 cm^2	The mouse and randomly selected keys on the keyboard were swabbed
Consulting table	Sponge swab	~5 cm^2	A sampling area was selected at random from the surface of the table
Dog washing table	Sponge swab	~5 cm^2	A sampling area was selected at random from the surface of the table
Door handles	Sponge swab	Front and back of handle	The front and back of the door handle was swabbed
Drinking fountain water tray	Wastewater	500 µL	The water in the tray was gently mixed to ensure an even suspension, then the sample was taken using a Pasteur pipette
Intubation/oxygen tube	Sponge swab	~5 cm^2	A sampling area was selected at random from the surface of a randomly selected intubation tube
Kennel waste drain	Sponge swab	500 µL	The sample was taken using a Pasteur pipette, close to where the drain exited the room (so all kennels would have drained through the sample site)
Outside open water pipe drain	Gel sample	500 µL	Wastewater was removed using a Pasteur pipette from the grate above the water line
Petroleum jelly	Wastewater	~500 µL	A Pasteur pipette was used to remove the petroleum jelly from the container. Due to its viscous nature

			and poor water solubility (it did not mix with the DNA/RNA shield), the sample size was approximate
Seating area	Sponge swab	~5 cm^2	A sampling area was selected at random from the surface of the benches
Sink plug holes	Sponge swab	500 µL	We did not have permission to remove the U-bends, so 500 µL wastewater was removed using a Pasteur pipette from the plug hole above
Table and equipment (dental theatre)	Sponge swab	~5 cm^2	A sampling area was selected at random from the surface of the table, as well as swabbing the switches that control the table and equipment
Thermometer	Sponge swab	Front and back of thermometer	The front and back of the thermometer was swabbed
Toilet flush	Sponge swab	Top and bottom of flush handle	The top and bottom of flush handle was swabbed
Ultrasound gel	Sponge swab	~500 µL	A Pasteur pipette was used to remove the ultrasound gel from the container. The sample was then mixed with DNA/RNA Shield in a screw cap tube
Ultrasound probe	Wastewater	~5 cm^2 of probe surface	The front, back and surface of the probe itself was swabbed
Waste pipe and water catch tray	Wastewater	500 µL	Wastewater was removed using a Pasteur pipette from the waste pipe water catch tray
Water in mop bucket	Sponge swab	500 µL	The water in the bucket was gently mixed to ensure an even suspension, then the sample was taken using a Pasteur pipette

4. Commonly seen resistances for the clinical species seen in this study

**Species**	**Commonly seen resistances**
Coagulase negative staph	Penicillin, oxacillin, gentamicin (80)
Enterococcus faecalis	Cephalosporins, lincosamides, clindamycin, aminoglycosides and some synthetic β-lactams (81)
Enterococcus spp	Cephalosporins, aminoglycosides, trimethoprim-sulfamethoxazole, Clindamycin, Lincosamides, Penicillinase-resistant penicillins, Bacitracin (81)
Escherichia coli	Tetracycline, sulfonamides, streptomycin, and ampicillin. *E. coli* found in animal hosts may also show resistances to phenicols, trimethoprim and Fosfomycin (82)
Hafnia alvei	Penicillin, oxacillin, amoxicillin plus clavulanic acid, colistin, narrow- and extended-spectrum cephalosporins (83,84)
Klebsiella oxytoca	Fluoroquinolones, erythromycin, tetracycline, chloramphenicol, cefepime (85,86)

Klebsiella pneumoniae	Ampicillin and some cephalosporins (87)
Moraxella group	vancomycin and trimethoprim. *M. catarrhalis* commonly produces a β lactamase (88,89)
Morganella morganii	Oxacillin, Ampicillin, Amoxicillin, most first- and second-generation cephalosporins, Macrolides, Lincosamides, Glycopeptides, Fosfomycin, Fusidic acid, Colistin (90,91)
Pasteurella multocida	*P. multocida* is generally thought to be susceptible to the majority of antimicrobials (92)
Pleuralibacter gergoviae	Penicillins, macrolides, lincosamides, streptogramins, rifampicin, fusidic acid, Fosfomycin, cefoxitin (likely due to β-lactamase production) (93,94)
Proteus mirabilis	Tetracycline, nitrofurantoin, polymyxin, colistin, reduced susceptibility to imipenem (95)
Pseudomonas aeruginosa	β-lactam and penem antibiotics (96)
Salmonella spp	Resistance to antimicrobials varies between *Salmonella* serotypes (97)
Serratia fonticola	Amoxicillin, ticarcillin (98)
Staphylococcus aureus	Methicillin-sensitive Staphylococcus aureus (MSSA) or methicillin-resistant Staphylococcus aureus (MRSA), which is resistant to penicillin, methicillin, aminoglycosides (99)
Staphylococcus haemolyticus	Noted to be one of the most resistant CoNS. Penicillins, cephalosporins, macrolides, quinolones, tetracyclines, aminoglycosides, glycopeptides, fosfomycin (100,101)
Staphylococcus lentus	Macrolides, lincosamides, streptogramins, tetracycline (102,103)
Staphylococcus pseudintermedius	Penicillin, tetracycline, erythromycin, amikacin, gentamicin, enrofloxacin (104,105)
Staphylococcus saprophyticus	Novobiocin (106)
Staphylococcus schleiferi	Penicillin, fluoroquinolones, methicillin (107)
Streptococcus canis	Some strains have shown resistance to vancomycin, linezolid. Further strains may also show resistance to β-lactams (108,109)

5. Supplementary figure 1

Total number of sequencing reads obtained from metagenomic sequencing stratified by A) location within the veterinary practice B) usage type and C) sample type. Y axis formatted to log 10.

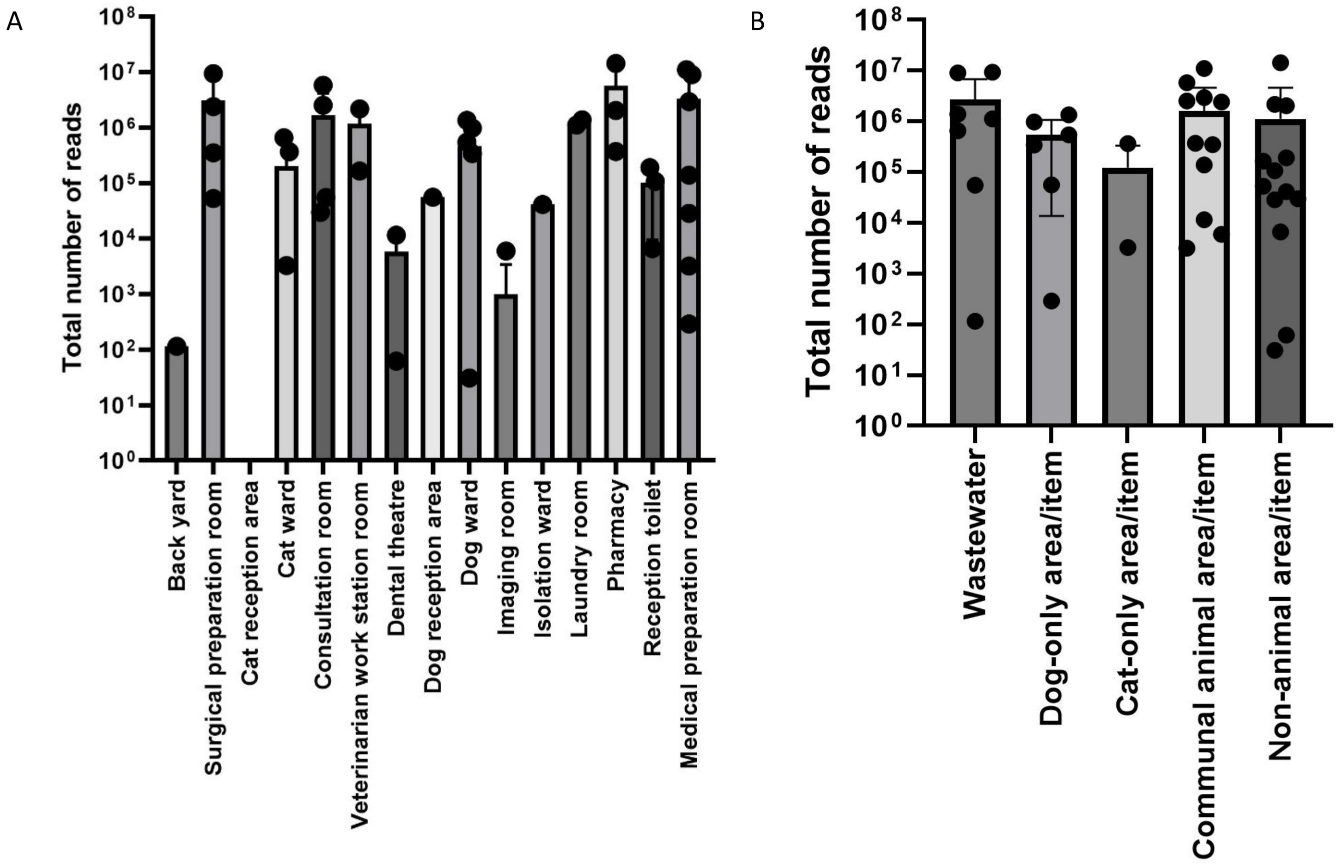

A
B
Total number of reads
10⁰
10¹
10²
10³
10⁴
10⁵
10⁶
10⁷
10⁸
Back yard
Surgical preparation room
Cat reception area
Cat ward
Consultation room
Veterinarian work station room
Dental theatre
Dog reception area
Dog ward
Imaging room
Isolation ward
Laundry room
Pharmacy
Reception toilet
Medical preparation room
Wastewater
Dog-only area/item
Cat-only area/item
Communal animal area/item
Non-animal area/item

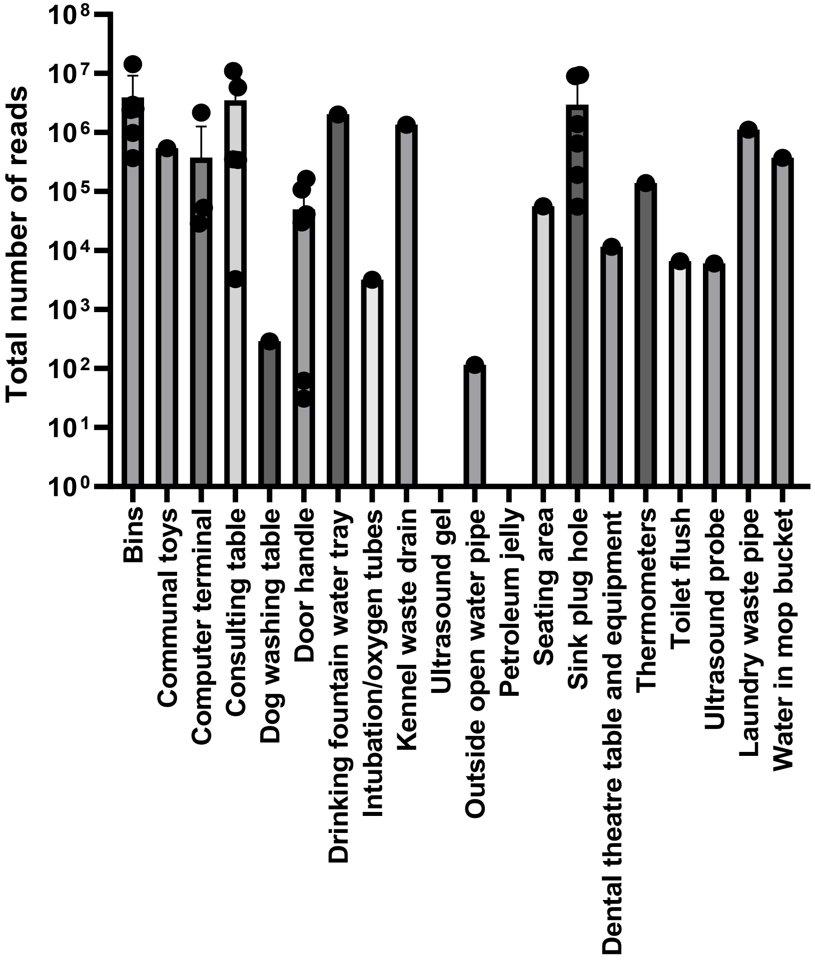

Total number of reads
10^8
10^7
10^6
10^5
10^4
10^3
10^2
10^1
10^0
Bins
Communal toys
Computer terminal
Consulting table
Dog washing table
Door handle
Drinking fountain water tray
Intubation/oxygen tubes
Kennel waste drain
Ultrasound gel
Outside open water pipe
Petroleum jelly
Seating area
Sink plug hole
Dental theatre table and equipment
Thermometers
Toilet flush
Ultrasound probe
Laundry waste pipe
Water in mop bucket

6. Phenotypic resistances from clinical isolates. I=intermediate resistance, NT=not tested, ICR=inducible clindamycin resistance, INN=innate resistance, EB=Enterobacteriaceae, NF=non-fermenter, NEB=non-Enterobacteriaceae, GN=Gram-negative, GP=Gram-positive, [1.] Low-level resistance (LLR) to aminoglycosides. Blue indicates an antibiotic was not tested for that bacterial species, white indicates 0% resistance, pink to red indicates increasing numbers of isolates with resistance to each antibiotic. Orange indicates the number of isolates with only intermediate resistance to each antibiotic. Yellow indicates the number of isolates with resistance and also intermediate resistance.

		Species	Total isolates	Gentamicin	Neomycin	Amikacin	Azithromycin	Ampicillin	Amoxicillin-clavulanic acid	Amoxicillin	Cloxacillin	Oxacillin	Penicillin	Cefovecin	Cephalexin	Cefpodoxime	ESBL screen	Chloramphenicol	Florfenicol
				Aminoglycocides				β lactams									ESBL screen	Amphenicols	
		Coagulase negative Staphylococci	4	0%	0%	100%	0%	25%	0%	25%	0%	0%	25%	0%	0%	NT	NT	0%	0%
		Enterococcus faecalis	12	INN[1]	INN[1]	INN[1]	INN[1]	0%	0%	0%	NT	NT	0%	INN	INN	INN	NT	0%	0%
		Enterococcus spp.	1	NT	NT	NT	NT	NT	0%	NT	NT	NT	0%	100%	100%	NT	NT	0%	0%
		Staphyococcus aureus	4	50%	0%	100%	25%	100%	50%	100%	50%	50%	100%	50%	50%	NT	NT	25%	0%
GP	GP	*Staphylococcus haemolyticus*	1	0%	0%	100%	0%	100%	100%	100%	100%	100%	100%	100%	0%	NT	NT	100%	0%
		Mammaliicoccus lentus	1	0%	NT	0%	0%	100%	100%	100%	100%	100%	100%	100%	100%	100%	NT	0%	0%
		Staphylococcus pseudintermedius	28	7% (I)	14% (2% I)	0%	29%	79%	14%	82%	14%	14%	82%	14%	14%	0%	NT	18%	0%
		Staphylococcus saprophyticus	1	0%	NT	0%	100%	100%	100%	100%	100%	100%	100%	100%	100%	100%	NT	0%	0%
		Staphylococcus schleiferi	4	0%	0%	0%	0%	0%	0%	0%	0%	0%	0%	0%	0%	NT	NT	0%	0%
		Streptococcus canis	6	INN[1]	INN[1]	INN[1]	INN[1]	0%	NT	NT	NT	NT	0%	NT	NT	NT	NT	0%	NT
		Escherichia coli	46	0%	2%	0%	NT	33%	17% (17% I)	33%	NT	NT	NT	13%	9%	9%	2%	4% (17% I)	NT
		Hafnia alvei	1	0%	0%	0%	NT	INN	INN	INN	NT	NT	NT	100% (I)	100%	100%	NT	0%	NT
		Klebsiella oxytoca	1	0%	0%	0%	NT	INN	0%	INN	NT	NT	NT	0%	0%	0%	0%	0%	NT
GN	EB	*Klebsiella pneumoniae*	3	0%	0%	0%	NT	INN	100%	INN	NT	NT	NT	0%	33%	0%	0%	0%	NT
		Morganella morganii	1	0%	0%	0%	NT	INN	INN	INN	NT	NT	NT	NT	100%	NT	NT	0%	NT
		Proteus mirabilis	7	0%	0%	0%	NT	43%	100% (I)	43%	NT	NT	NT	14% (I)	14%	0%	NT	14% (14% I)	NT
		Salmonella spp.	1	NT	0%	NT	NT	0%	0%	0%	NT	NT	NT	0%	NT	0%	NT	0%	NT
		Moraxella group	1	0%	NT	0%	NT	0%	0%	NT	NT	NT	NT	INN	NT	NT	NT	0%	NT
GN	NEB	*Pasteurella multocida*	10	NT	0%	NT	NT	0%	0%	0%	NT	NT	NT	0%	NT	NT	NT	NT	NT
		Pluralibacter gergoviae	1	0%	0%	0%	NT	NT	0%	NT	NT	NT	NT	0%	100%	0%	NT	100% (I)	NT
		Serratia fonticola	1	0%	0%	0%	NT	NT	0%	NT	NT	NT	NT	0%	0%	NT	NT	0%	NT
GN	NF	*Pseudomonas aeruginosa*	13	0%	INN	0%	NT	INN	INN	INN	NT	NT	NT	NT	NT	NT	NT	INN	INN

				Fluoroquinolones					Fluorfenicols	Fusidic acid	Lincosamides		Macrolide	Sulphonamides
		Species	Total isolates	Ciprofloxacin	Enrofloxacin	Marbofloxacin	Moxifloxacin	Pradofloxacin	Florfenicol	Fusidic acid	Clindamycin	Lincomycin	Erythromycin	Potentiated sulphonamides
GP	GP	Coagulase negative Staphylococci	4	0%	0%	0%	NT	0%	0%	0%	0%	0%	0%	0%
		Enterococcus faecalis	12	25% (I)	25% (I)	17% (17% I)	NT	NT	0%	NT	INN	NT	INN	INN
		Enterococcus spp.	1	0%	0%	0%	NT	NT	0%	NT	NT	NT	NT	NT
		Staphyococcus aureus	4	0%	0%	0%	NT	25%	0%	0%	25%	0%	25%	50%
		Staphylococcus haemolyticus	1	0%	0%	0%	NT	0%	0%	100%	0%	0%	0%	0%
		Staphylococcus lentus	1	0%	0%	0%	NT	0%	0%	NT	NT	NT	0%	0%
		Staphylococcus pseudintermedius	28	7%	7%	7%	NT	7%	0%	12%	33%	29%	29%	21%
		Staphylococcus saprophyticus	1	0%	0%	0%	NT	0%	0%	INN	NT	NT	100%	0%
		Staphylococcus schleiferi	4	25%	25%	25%	NT	25% (I)	0%	25%	0%	0%	0%	0%
		Streptococcus canis	6	NT	NT	NT	0%	NT	NT	INN	0%	NT	0%	0%
GN	EB	*Escherichia coli*	46	4% (4% I)	4% (4% I)	4%	NT	4% (2% I)	NT	NT	NT	NT	NT	13%
		Hafnia alvei	1	0%	0%	0%	NT	0%	NT	NT	NT	NT	NT	0%
		Klebsiella oxytoca	1	0%	0%	0%	NT	0%	NT	NT	NT	NT	NT	0%
		Klebsiella pneumoniae	3	0%	0%	0%	NT	0%	NT	NT	NT	NT	NT	0%
		Morganella morganii	1	0%	0%	0%	NT	0%	NT	NT	NT	NT	NT	0%
		Proteus mirabilis	7	0%	0%	0%	NT	0%	NT	NT	NT	NT	NT	14%
		Salmonella spp.	1	0%	0%	0%	NT	0%	NT	NT	NT	NT	NT	0%
GN	NEB	Moraxella group	1	0%	0%	0%	NT	NT	NT	NT	NT	NT	NT	0%
		Pasteurella multocida	10	0%	0%	0%	NT	0%	NT	NT	NT	NT	NT	0%
		[illegible]	[illegible]	[illegible]	[illegible]	[illegible]	[illegible]	[illegible]	[illegible]	[illegible]	[illegible]	[illegible]	[illegible]	[illegible]

		Species	Total isolates	Tetracyclines: Doxycycline	Tetracyclines: Minocycline	Tetracyclines: Tetracycline	Mupirocin: Mupirocin	Nitrofurantoin: Nitrofurantoin	Rifamycins: Rifampicin	Lincosamides: Clindamycin	Lincosamides: Lincomycin	ICR test: ICR test	Macrolide: Erythromycin	Sulphonamides: Potentiated sulphonamides	Tetracyclines: Doxycycline	Tetracyclines: Minocycline	Tetracyclines: Tetracycline	Nitrofurantoin: Nitrofurantoin	Rifamycins: Rifampicin
		Coagulase negative Staphylococci	4	NT	0%	NT	0%	NT	0%	0%	0%	NT	0%	0%	NT	0%	NT	NT	0%
		Enterococcus faecalis	12	17%	17%	NT	NT	0%	NT	NT	NT	NT	NT	NT	17%	17%	NT	0%	NT
		Enterococcus spp.	1	0%	0%	NT	NT	100% (I)	NT	NT	NT	NT	NT	NT	0%	0%	NT	100% (I)	NT
		Staphyococcus aureus	4	NT	25%	NT	0%	NT	0%	25%	0%	25%	25%	50%	NT	25%	NT	NT	0%
GP	GP	*Staphylococcus haemolyticus*	1	NT	0%	NT	0%	NT	0%	0%	0%	0%	0%	0%	NT	0%	NT	NT	0%
		Staphylococcus lentus	1	0%	0%	NT	NT	0%	NT	NT	NT	NT	0%	0%	0%	0%	NT	0%	NT
		Staphylococcus pseudintermedius	28	50%	0%	NT	0%	0%	0%	33%	29%	22%	29%	21%	50%	0%	NT	0%	0%
		Staphylococcus saprophyticus	1	100%	0%	NT	NT	0%	NT	NT	NT	NT	100%	0%	100%	0%	NT	0%	NT
		Staphylococcus schleiferi	4	NT	0%	NT	0%	NT	0%	0%	0%	0%	0%	0%	NT	0%	NT	NT	0%
		Streptococcus canis	6	0%	NT	17%	NT	NT	NT	0%	NT	NT	0%	0%	0%	NT	17%	NT	NT
		Escherichia coli	46	15%	NT	15%	NT	0%	NT	NT	NT	NT	NT	13%	15%	NT	15%	0%	NT
		Hafnia alvei	1	0%	NT	0%	NT	0%	NT	NT	NT	NT	NT	0%	0%	NT	0%	0%	NT
		Klebsiella oxytoca	1	0%	NT	0%	NT	0%	NT	NT	NT	NT	NT	0%	0%	NT	0%	0%	NT
GN	EB	*Klebsiella pneumoniae*	3	0%	NT	0%	NT	66% (I)	NT	NT	NT	NT	NT	0%	0%	NT	0%	66% (I)	NT
		Morganella morganii	1	INN	INN	INN	NT	INN	NT	NT	NT	NT	NT	0%	100%	NT	100%	100%	NT
		Proteus mirabilis	7	INN	INN	INN	NT	INN	NT	NT	NT	NT	NT	14%	100%	NT	100%	100%	NT
		Salmonella spp.	1	0%	NT	0%	NT	NT	NT	NT	NT	NT	NT	0%	0%	NT	0%	NT	NT
		Moraxella group	1	0%	NT	0%	NT	NT	NT	NT	NT	NT	NT	0%	0%	NT	0%	NT	NT
GN	NEB	*Pasteurella multocida*	10	NT	NT	0%	NT	NT	NT	NT	NT	NT	NT	0%	NT	NT	0%	NT	NT
		Pluralibacter gergoviae	1	0%	NT	0%	NT	0%	NT	NT	NT	NT	NT	0%	0%	NT	0%	0%	NT
